# Impact of microencapsulated *Ziziphora tenuior* essential oil and orange fiber as natural‐functional additives on chemical and microbial qualities of cooked beef sausage

**DOI:** 10.1002/fsn3.2943

**Published:** 2022-05-30

**Authors:** Majid Aminzare, Mohammad Hashemi, Asma Afshari, Mohammad Hossein Mokhtari, Seyyed Mohammad Ali Noori

**Affiliations:** ^1^ Department of Food Safety and Hygiene, School of Public Health Zanjan University of Medical Sciences Zanjan Iran; ^2^ Medical Toxicology Research Center Mashhad University of Medical Sciences Mashhad Iran; ^3^ Department of Nutrition, Faculty of Medicine Mashhad University of Medical Sciences Mashhad Iran; ^4^ Department of Food and Drug Control, School of Pharmacy Ahvaz Jundishapur University of Medical Sciences Ahvaz Iran; ^5^ Toxicology Research Center, Medical Basic Sciences Research Institute Ahvaz Jundishapur University of Medical Sciences Ahvaz Iran; ^6^ Department of Nutrition, School of Allied Medical Sciences Ahvaz Jundishapur University of Medical Sciences Ahvaz Iran

**Keywords:** natural preservative, orange fiber, sausage, shelf life, *Ziziphora tenuior* essential oil

## Abstract

The aim of the current study was to investigate the suitability of *Ziziphora tenuior* essential oil (ZEO) as a preservative. For this purpose, the effect of free and microencapsulated ZEO, combined with orange fiber, was determined on the chemical and microbial qualities of cooked beef sausage. In this study, modified starch was used for encapsulation of essential oil, and subsequently, 0.5% ZEO and 1% orange fiber were used for preparing cooked beef sausages during 60 days of storage at 4°C. To assess the microbial quality of samples, total viable count (TVC), psychrophilic count (PSY), and lactic acid bacteria (LAB) were analyzed. Furthermore, peroxide value (PV) and thiobarbituric acid reactive substances (TBARS) were tested to examine lipid oxidation. The most components of ZEO were pulegone (47.12%), isomenthone (14.57%), and 1,8‐cineole (12.84%) according to GC–MS analysis. The reducing power, DPPH radical scavenging activity, MIC, and MBC of ZEO were 16.44 (EC_50_), 8.36 (IC_50_), 0.625–2.5, and 1.25–5 mg/ml, respectively. Moreover, sausage containing 0.5% microencapsulated ZEO in combination with 1% orange fiber showed the best results with the following values (*p* ≤ .05): TVC (3.69 log CFU/g), PSY (3.51 log CFU/g), LAB (3.1 log CFU/g), PV (10.41 meq/kg lipid), and TBARS (3.1 mg MDA/kg). This is due to the antimicrobial and antioxidant properties of microencapsulated essential oil. Therefore, the results of the present study can be applied in the meat industries as a new natural preservation method.

## Graphical abstract

The current paper concerned with the simultaneous use of microencapsulated essential oils and fiber in meat sausage samples. combination of essential oil and fiber significantly increased the shelf life and quality indices of sausages.

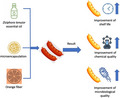

## 1 INTRODUCTION

Meat and meat products, such as sausages, deem as important food products with a great deal of socioeconomic impacts. Regrettably, meat products are categorized as perishable foods due to their rapid spoilage and safety challenges. The main factors causing spoilage of sausage and reducing its shelf life are microbial growth and lipid oxidation (Luong et al., 2020; Zehi et al., 2020). Microbial contamination of meat and meat products is frequently reported by legal authorities (EFSA & ECDC, 2019). In addition, lipid oxidation is regarded as one of the most important criteria for meat and meat product quality (Bolívar‐Monsalve et al., 2019). In recent years, in order to control the spoilage of these products, the formulation and production of novel processed meat, with functional properties and without chemical preservatives, has been one of the main priorities for the research and development section of meat industries to respond to the green marketing and consumerism demand (Alirezalu et al., 2021; Pereira et al., 2019).

Essential oils (EOs) are natural compounds, proved to have antimicrobial and antioxidant activity (Mohajer et al., 2021), and have been widely used in processed meats due to their beneficial effects (Šojić et al., 2021; Tomović et al., 2020). Addition of Eos to sausages formulation improves their microbial and chemical stability and safety (Šojić et al., 2021; Viuda‐Martos et al., 2010a). Microencapsulation of Eos has progressed remarkably in recent years and could provide several benefits, including controlled release, reduced volatility, increased availability, solubility, stability, and bioavailability (increased antimicrobial and antioxidant activities; Abd Manaf et al., 2015; Viacava et al., 2018). Encapsulation of EOs can increase the durability and antimicrobial and antioxidant strengths of EOs in the sausage samples due to the slow release of EOs (Ji et al., 2022; Vafania et al., 2019).

*Ziziphora tenuior* is a plant belonging to the *Lamiaceae* family, and its EO is recognized as an aromatic compound with remarkable antioxidant and antimicrobial activities (Behravan et al., 2007). The antimicrobial properties of *Z. tenuior* have been investigated against *Escherichia coli*, *Staphylococcus aureus*, and *Shigella dysenteriae* (Mahboubi et al., 2014), as well as its antioxidant properties (Dakah et al., 2014).

Different dietary fibers have been added to food formulas, as an ingredient, to produce new functional foods such as meat products (Díaz‐Vela et al., 2017), chicken nuggets (Mohd Zaini et al., 2021), and dairy products (Yi et al., 2014). The advantages of the addition of fibers to foods can be divided into two categories: (a) promote foods' properties such as improving the texture and color, reducing sugar and fat content, and enhancing antioxidant activity; (b) beneficial effects on human health including cardiovascular health and weight management (Viuda‐Martos et al., 2010b). Herein, the addition of fiber to foods has become an upward trend in the market (Sloan, 2000).

Various researches have investigated the use of the free form of different EOs in combination with different fibers on the chemical and microbial stability of meat and meat products (Aminzare et al., 2018; Araújo et al., 2018; Bianchin et al., 2017; da Silva et al., 2018; Huang et al., 2021; Meira et al., 2017; Mohammadpourfard et al., 2021; Viuda‐Martos et al., 2010a, 2010b), while as far as we know, *there* are *no* previous *studies* evaluating antimicrobial and antioxidant effects of EOs, in microencapsulated and free forms, in combination with fibers. Therefore, the objectives of the current work were to (a) examine the total phenolic content and chemical composition of ZEO, (b) determine the in vitro antimicrobial and antioxidant activities of ZEO, and (c) evaluate the effects of ZEO, in free and microencapsulated forms, alone and in combination with orange fiber on microbial and oxidative stability of cooked beef sausage stored at 4°C during 60 days.

## 2 MATERIALS AND METHODS

### 2.1 Chemicals and reagents

Sodium carbonate, Folin–Ciocalteu reagent, gallic acid standard, potassium ferricyanide, ferric chloride, 2,2‐diphenyl‐1‐picrylhydrazyl (DPPH), butylated hydroxytoluene (BHT), phosphate buffer, ethanol, trichloroacetic acid, and methanol were purchased from Sigma‐Aldrich (St. Louis., MO, USA). Chloroform, acetic acid, potassium iodide, sodium thiosulfate, thiobarbituric acid (TBA), perchloric acid, dimethyl sulfoxide (DMSO), ampicillin, nutrient agar, Brain Heart Infusion (BHI) agar, BHI broth, de Man Rogosa Sharpe (MRS), plate count agar (PCA), ampicillin, and GasPak system type C were purchased from Merck (Darmstadt, Germany). Orange fiber and filter paper were provided from FiberStar (River Falls, WI, USA) and Whatman International, respectively (Maidstone, UK).

### 2.2 Extraction of essential oil

*Ziziphora tenuior* herb was gathered from Zanjan province, Iran (spring 2020), and a senior taxonomist confirmed the plant species. Cold tap water was used to wash the aerial section of the herb and placed at 25°C to become dry. A mixer device (ParsKhazar, Tehran, Iran) was used to ground dry herbs. By using a Clevenger apparatus (KOL, behr, Düsseldorf, Germany) ZEO was extracted (hydrodistillation method). The extraction process was continued for about 180 min at 100°C. Sodium sulfate was used for dehydration of oil and subsequently filtered through a 0.22 μm filter. Yielded EO was transferred to a dark, sealed glass container and placed at 4°C away from direct light, until use (Hamedi et al., 2017).

### 2.3 ZEO gas chromatography–mass spectrometry (GC–MS) analysis

A Hewlett Packard 5890 device (using HP‐5MS column; the dimension of the film was 30 × 0.25 mm ID × 0.25 mm) was used to analyze the chemical composition of ZEO. For the beginning, the device temperature was set at 50°C; it increased 2°C per min to reach 120°C and remained at 120°C for 3 min. The temperature was raised to 300°C, and the helium flow rate during this process was 1 ml/min. Also, ionization energy was 70 eV for the MS process. Subsequently, retention indices were compared with samples presented by the library (Wiley‐VCH2001 data software, Weinheim, Germany).

### 2.4 Analysis of total phenolic content

Folin–Ciocalteu reagent assay was used to measure the amount of total phenolic compounds of ZEO (Aliakbarlu et al., 2013; Singleton et al., 1999). An amount of 2.25 ml of distilled water and 500 μl of EO in methanol mixture (4 mg/ml) were transferred to a glass tube. Folin–Ciocalteu reagent (250 μl) was then added to the tube and mixed vigorously. Two milliliters of sodium carbonate solution (7.5%; w/v) was added and mixed, 5 min after the addition of the reagent. The tube was then placed at 25°C for 120 min, and by using a spectrophotometer (CECIL, Cambridge, UK), at 760 nm, absorbance was recorded. Five hundred microliter 50% (v/v) methanol was used as blank. The same procedure was also applied to a standard solution of gallic acid, and a standard curve was obtained. The content of total phenolic was shown as mg GAE/g oil.

### 2.5 Determination of in vitro antioxidant and antimicrobial activities of ZEO

#### 2.5.1 Antioxidant properties of ZEO

##### DPPH free radical scavenging activity

The DPPH free radical scavenging method was used to investigate the antioxidant properties of ZEO and reported as IC_50_ values. For this purpose, 1 ml of ZEO with different concentrations (0.312–10 mg/ml) was transferred separately to a glass tube and DPPH methanolic solution (1 ml, 90 μM). Methanol was added to the tube to reach the final volume of 4 ml. The glass tube was shaken well and incubated at room temperature for 1 h, away from direct light. Using a spectrophotometer (CECIL) at 517 nm, the absorbance was measured against a blank. For each concentration, radical scavenging activity was figured by the following equation:

$$\text{Radical scavenging activity}\;\left(\%\right)=\left({\mathrm{Abs}}_{\text{DPPH}}-{\mathrm{Abs}}_{\text{sample}}\right)/{\mathrm{Abs}}_{\text{DPPH}}\times 100.$$

Abs_DPPH_: methanolic solution of DPPH absorbance

Abs_sample_: ZEO absorbance

In this test, the positive control was BHT. The concentration of the sample was represented by IC_50_ values. Based on the graph, for IC_50_, it was needed to scavenge half of DPPH free radicals, which was plotted for inhibition percentage (Guleria et al., 2013).

##### Ferric (Fe3+) reducing power

*Ziziphora tenuior* essential oil was analyzed to determine its ferric reducing power introduced by Guleria et al. (2013). Potassium ferricyanide (2.5 ml, 1%) and phosphate buffer (2.5 ml, 0.2 M, pH 6.6) were added to 1 ml of each ZEO concentrations (0.312–10 mg/ml) in a glass tube and placed in an incubator at 50°C for 20 min. Subsequently, trichloroacetic acid (2.5 ml, 10%) was transferred to the glass tube. Centrifugation of the mixture was carried out for 10 min at 1000 *g*. The supernatant (2.5 ml) was transferred to another tube, 2.5 ml ferric chloride solution (1%) and 2.5 ml distilled water were mixed with supernatant, and the absorbance of tube solution was recorded with a double beam ultraviolet‐VIS spectrophotometer at 700 nm. Higher reducing power displays higher absorbance. The graph of absorbance was used for the calculation of 0.5 of absorbance (EC_50_). EC_50_ of ZEO compared with standard antioxidant and positive control in this analysis was BHT.

#### 2.5.2 Antimicrobial properties of ZEO

##### Preparation of bacteria

In this study, 10 common foodborne pathogens (*Listeria monocytogenes* (PTCC 1299), *Bacillus subtilis* (PTCC 1204), *Salmonella typhimurium* (PTCC 1709), *Staphylococcus aureus* (PTCC 1917), *Bacillus cereus* (PTCC 1539), *Staphylococcus epidermidis* (PTCC 1856), *Enterobacter faecalis* (ATCC 29212), *Klebsiella pneumoniae* (PTCC 1290), *Shigella dysenteriae* (PTCC 1188), and *Escherichia coli* (PTCC 1769) were selected and obtained from the IROST (Tehran, Iran). Fifteen milliliters of BHI broth was used to activate bacteria (24 h, at 37°C; Ojagh et al., 2010). Centrifugation was then carried out, and bacterial cells were washed two times with normal saline. The concentration of each bacterium was adjusted by a spectrophotometer (CECIL) at 600 nm to the required density (~1.5 × 10^8^ CFU/ml) and dilution was carried out to achieve proper bacterial density (~1.5 × 10^6^ CFU/ml) for further evaluation. For confirmation of the results, bacteria were cultivated in BHI agar and placed in an incubator for 24 h at 37°C.

##### Agar disk diffusion

By using the agar disk diffusion method, ZEO was analyzed for antimicrobial activity against 10 aforementioned bacteria (Goni et al., 2009). Briefly, 100 μl of overnight grown bacteria (~1.5 × 10^6^ CFU/ml) was cultivated on nutrient agar. Sterile filter paper in the shape of disks, containing 10 μg/disk of ZEO, was then placed onto the surface of culture media and were placed into an incubator for 24 h at 37°C, to allow bacterial growth. Blank disk (with no antibacterial agent) and disks containing ampicillin (10 μg/disk) were used as negative and positive controls, respectively. Growth inhibition of bacteria was determined by measuring the inhibition zone diameters of surrounding disks by caliper (Zaidan et al., 2005).

##### Minimum inhibitory concentration (MIC) and minimum bactericidal concentration (MBC)

The MIC values for ZEO were investigated according to the study of Aliakbarlu et al. (2013). Briefly, 20 μl of various concentrations of ZEO (3.125–100 mg/ml) in DMSO 5% as well as 20 μl of each bacterial suspension and 160 μl of BHI broth were transferred into 96‐well microplates. The final concentration of ZEO was in the range of 0.312–10 mg/ml; each well was reached to 200 μl final volume, containing ~1.5 × 10^5^ CFU/ml bacteria. Positive control had culture medium and suspension of bacteria and the negative control contained the medium and ZEO. A microplate shaker was used to mix the plates (for about 30 s, at 300  *g*) and finally placed in an incubator for 24 h at 37°C. The minimum concentration of ZEO with no bacterial growth (without turbidity) was considered as MIC. Wells with no visible growth were then cultured on BHI agar and placed in an incubator at 37°C for 24 h. The 99.9% bacterial death caused by the lowest ZEO concentration was considered as MBC (Araújo et al., 2018).

### 2.6 Preparation of microencapsulated ZEO

*Ziziphora tenuior* essential oil was microencapsulated according to the method previously described by Baranauskienė et al. (2007)) with some modifications. Modified starch (30% w/w) was added to 40°C deionized water and dispersed. The solution was then cooled and mixed for 12 h to increase hydration. *Ziziphora tenuior* essential oil (10% w/w) was added to hydrated coating starch and emulsified. An Ultra Turrax homogenizer (IKA T25 basic, Staufen, Germany) was utilized for homogenization, functioned at 13,500 *g* for 7 min. The emulsion was spray‐dried in a mini spray dryer (Buchi B191, Flawil, Gallen, Switzerland) under the following parameters: The emulsion inlet flow was 15 ml/min and inlet and outlet temperature of air were 180 ± 10 and 90 ± 10°C, respectively. Yielded powder was placed at −18°C freezer until use.

### 2.7 Sausage formulation

The sausage was prepared in the Dara meat industry, Tehran, Iran. Mortadella‐type sausage was produced according to a traditional and commercial formula using 50% beef meat, 18% ice, 16% vegetable oil, 10% flour, 3% potato starch, 1.2% sodium chloride, 0.7% garlic, 0.6% spice mix (black pepper, nutmeg, and ginger), 0.05% sodium ascorbate, 0.012% sodium nitrate, and 0.5% sodium tripolyphosphate. They were then subjected to the cutter and homogenized thoroughly. The batter was then divided into five equal portions, and experimental groups were prepared using free or microencapsulated ZEO (the final concentration in the product was 0.5% w/w of pure ZEO) alone and in combination with 1% (w/w) orange fiber (Table 1). Herein, the minimum effective concentration of ZEO and orange fiber was used in order to avoid undesirable sensory effects on sausage sample (Fernández‐López et al., 2008; Rezaei & Shahbazi, 2018). Subsequently, they were packed into polyamide bags (50 mm caliber; IranNavid, Tehran, Iran) weighed 50 ± 1 g, closed and sealed at both ends, placed in a cooking room, monitored by a thermocouple probe (Omega Engineering, Stamford, CT, USA), and ensured that sausage center reached to 75°C. The sausage was placed in a water bath to cool down and subsequently at 4°C until analysis. Analysis was carried out at 0, 10, 20, 40, and 60 days of storage.

**TABLE 1:** Different treatments and their abbreviations

Name	Treatment
CON	No treatment
ZEO	Cooked sausage consists of 0.5% (w/w) *Ziziphora tenuior* EO
MZEO	Cooked sausage containing 0.5% (w/w) microencapsulated *Ziziphora tenuior* EO
ZEOF	Cooked sausage consists of 0.5% (w/w) *Ziziphora tenuior* EO + 1% (w/w) Orange fiber
MZEOF	Cooked sausage containing 0.5% (w/w) microencapsulated *Ziziphora tenuior* EO + 1% (w/w) Orange fiber

Abbreviation: EO, essential oil.

### 2.8 Evaluation of microbial and chemical qualities of sausages

#### 2.8.1 Microbiological analysis

Major microbiological quality indicators of sausages were evaluated during the storage period. Total psychrophilic bacteria count (PSY), total viable count (TVC), and lactic acid bacteria (LAB) were investigated for the microbiological quality of sausages. Briefly, 90 ml sterile peptone water (0.1%) and 10 g of sample were placed in a sterile bag and homogenized using a stomacher (Interscience, St. Nom, France) for 1 min. Subsequently, serial dilutions were prepared in glass tubes, and diluted samples were transferred to appropriate culture media. The microbiological analysis was carried out with respect to the study of Esmaeili et al. (2020). For TVC, 0.1 ml of proper dilution was transferred to PCA medium and placed in an incubator for 24 h at 37°C. A similar method was used for PSY, but plates were stored for 10 days at 7°C. To perform LAB count, 1 ml of diluted samples was poured into sterile plates, and 15–20 ml of MRS agar was added to the plates. The plates were then gently rotated and incubated anaerobically (anaerobic jars with GasPak system type C) at 30°C for 72 h. The mean was reported as log CFU/g (Nisar et al., 2019).

#### 2.8.2 Determination of lipid oxidation

##### Peroxide value (PV)

The peroxide value of the extracted total lipid from sausage samples was analyzed, similar to the method previously illustrated by Lekjing (2016). Thirty milliliter chloroform: acetic acid (2:3) and 1 g lipid sample were added to a plastic container and shaken vigorously. Then, saturated potassium iodide solution (0.5 ml) was poured into the container and held at room temperature protected from light, for 1 min. Subsequently, distilled water (30 ml) was added to the container. The indicator (starch solution, 0.5 ml, 1% w/v) was transferred to the mixture and standardized. Sodium thiosulfate solution (0.01 N) was used for the titration of iodine. The values were expressed as meq/kg lipid.

##### Thiobarbituric acid reactive substances (TBARS)

Ten grams of beef sausage samples were homogenized with extraction solution (35 ml, containing 1 ml BHT [5 mg/ml] and 4% perchloric acid) for 1 min. The solution was filterate through a Whatman filter paper and collected in a 50 ml falcon tube. By adding 4% perchloric acid, the filtrate was reached to 50 ml, and TBA solution (5 ml, 0.02 mol/L) and the adjusted mixture (5 ml) was poured into a glass tube. Subsequently, the glass tube was then shaken by a vortex mixer (Lab Genius, London, UK) and placed in a water bath (100°C) for 1 h for the formation of malonaldehyde–TBA complex. A spectrophotometer (CECIL) was used to measure sample absorbance at 532 nm. The amounts of TBARS were shown as mg MDA/kg. TEP solution was utilized to prepare standard curve (Pikul et al., 1989).

### 2.9 Statistical analysis

In this study, the entire tests were performed in technical triplicates, and the results were statistically analyzed using SPSS software (SPSS Statistics Software, version 18). The data were tested by using one‐way ANOVA and Tukey's test. *p* ≤ .05 was considered as a statistical significance level.

## 3 RESULTS AND DISCUSSION

### 3.1 ZEO chemical analysis

The components of essential oils exhibit antimicrobial and antioxidant properties, and analysis of Eos reveals useful information about their beneficial effects on the shelf life of food. Major constituents of ZEO are depicted in Table 2. The GC–MS analysis was detected 23 compounds in essential oil, and it consisted 96.47% of total EO. The main components of ZEO were pulegone (47.12%), isomenthone (14.57%), 1,8‐cineole (12.84%), and piperitenone (9.31%), respectively. Pulegone was reported as the major component of ZEO by other studies, which was in accordance with the current study (Baser et al., 1991; Behravan et al., 2007; Ozturk & Ercisli, 2007). However, some other main components reported were different; for example, in the study of Behravan et al. (2007), other main constituents of ZEO were terpineol (14.5%) and methyl acetate (10.9%). Besides, Ajourloo et al. (2021) reported geraniol (20.62%) and carvacrol (18.17%) as the major constituents for ZEO, which was inconsistent with the current study. These variations in the major components of EOs attributed to environmental factors, genetic factors such as cultivar, extraction method, maturity of the plants, the solvent used for extraction, geographical location, the part used of plant, the season of harvest, and cultivation conditions (Hazrati et al., 2020). Moreover, it has been proven that pulegone is effective against some bacteria, making it suitable for microbial growth in food models (Damani et al., 2022). Damani et al. (2022) reported antioxidant properties for pulegone and stated that EOs containing pulegone are an appropriate alternative for application in food instead of synthetic preservations. In addition, excellent antioxidant and antimicrobial effects of isomenthone have been reported (Alizadeh et al., 2021). Joshi et al. (2022) also reported significant antimicrobial properties of 1,8‐cineole against *S. aureus*, *E. coli*, and *Salmonella typhymurium*.

**TABLE 2:** *Ziziphora tenuior* chemical composition

Components	Retention index	Area (%)
α‐Pinene	975	1.16
p‐Cymene	1010	0.45
1,8‐Cineole	1018	12.83
y‐Terpinene	1052	1.04
Linalool	1076	0.71
Menthone	1132	0.13
Menthol	1167	0.58
Isomenthol	1182	0.29
Verbenone	1192	1.10
Pullegone	1214	47.12
Isomenthone	1217	14.54
Piperitone	1228	0.64
Carvone	1250	0.13
Thymol	1261	0.94
iso‐Isopulegol	1266	0.22
Terpinen‐4‐ol	1174	0.88
Carvacrol	1286	0.19
lsomenthyl acetate	1291	0.79
Eugenol	1318	1.40
Piperitenone	1325	9.31
B‐Bourbonene	1388	0.11
Germacrene	1427	1.36
Spathulienol	1558	0.55
Total		96.47

### 3.2 ZEO total phenolic content

Table 3 represents the total phenol content of ZEO. The amount of total phenols of ZEO was recorded 184 ± 4.21 mg GAE/g. Hazrati et al. (2020) reported 30.3 ± 0.1 mg GAE/g for *Z. tenuior*, which was significantly lower than the current study. However, Salehi et al. (2005) reported 174.8 ± 1.2 mg GAE/g for *Ziziphora clinopodioides* EO. This difference could be due to plant species, geographical location of the plant, the method of EO preparation, etc. It should be noted that higher amounts of phenolic content exhibit better antioxidant and antimicrobial activities (Hazrati et al., 2020). The high total phenol content of ZEO in our study indicates excellent radical scavenging properties. This phenomenon made the ZEO suitable for application in food models that are susceptible to oxidative spoilage, such as sausage products (Ajourloo et al., 2021).

**TABLE 3:** Antioxidant activities and total phenolic content of *Ziziphora tenuior* essential oil (mean ± SD)

Sample	DPPH IC_50_ (mg/ml)	Total phenolic content (mg GAE/g)	Reducing power EC_50_ (mg/ml)
ZEO	8.36 ± 0.21	184 ± 4.21	16.44 ± 0.78
BHT	0.58 ± 0.08	—	2.77 ± 0.12

### 3.3 In vitro antioxidant activities of ZEO

The results of antioxidant activities of ZEO were measured by reducing power and DPPH free radical scavenging methods, which are shown in Table 3. The required concentration of the antioxidant for scavenging 50% of DPPH radicals is defined as the IC_50_ value for DPPH scavenging activity. DPPH assay showed lower antioxidant power for ZEO in comparison with BHT. In fact, the recorded IC_50_ of the ZEO (8.36 mg/ml) was approximately 14 times higher than the value measured for BHT (0.58 mg/ml). In the study of Alp et al. (2016), antioxidant properties of *Z. clinopodioides* with different origins were investigated and the IC_50_ results were in the range of 3.6–4.2 mg/ml, which were lower compared to our study.

Since the effects of antioxidant compounds depend on diverse factors with multiple mechanisms of action, it is essential to investigate their in vitro antioxidant capacity by various methods (Pérez‐Jiménez et al., 2008). Therefore, in the present study, the in vitro antioxidant capacity of ZEO was also investigated by the reducing power method. The EC_50_ of ZEO reducing power in the current work was lower than the study of Sarikurkcu et al. (2019) about ferric reducing power of *Ziziphora taurica* EO. Antioxidant properties of plant EOs are related to the presence of bioactive components such as oxygenated monoterpenes and monoterpene hydrocarbons. The low antioxidant activity of ZEO in our study might be due to the low quantity of oxygenated monoterpenes such as thymol and carvacrol (Cao et al., 2009).

### 3.4 In vitro antibacterial activity of ZEO

In vitro antibacterial analysis of ZEO showed inhibition for growth patterns against pathogens (Tables 4 and 5). The disk diffusion method showed that *L. monocytogenes* was the most sensitive microorganism, followed by *S. epidermis* and *S. aureus*, respectively, while the most resistant pathogen was *K. pneumonia*. It should be mentioned that ZEO showed higher antibacterial activities than ampicillin, implying the effectiveness of ZEO as an antimicrobial agent. Nazemisalman et al. (2018) showed excellent antibacterial effects of ZEO, using the disk diffusion method, which was in accordance with the current study.

**TABLE 4:** Antibacterial properties of *Ziziphora tenuior* essential oil against some pathogenic bacteria (mean ± SD)

Bacteria	Diameter of inhibition zone (mm)	
	ZEO (10 μg/disk)	Ampicillin (10 μg/disk)
Gram‐positives		
*S. aureus*	25.97 ± 0.11^f^	15.97 ± 0.11^f^
*S. epidermis*	27.97 ± 0.58^g^	20.93 ± 0.61^g^
*L. monocytogenes*	31.03 ± 0.31^h^	25.90 ± 0.26^h^
*B. cereus*	23.97 ± 0.32^e^	11.07 ± 0.32^c^
*B. subtilis*	25.07 ± 0.61^ef^	13.03 ± 0.57^e^
*E. faecalis*	16.93 ± 0.11^b^	13.01 ± 0.10^e^
Gram‐negatives		
*E. coli*	21.00 ± 0.35^d^	12.03 ± 0.25^d^
*S. typhimurium*	19.03 ± 0.11^c^	9.03 ± 0.11^a^
*K. pneumonia*	15.13 ± 0.61^a^	10.07 ± 0.32^b^
*S. dysenteriae*	24.07 ± 0.30^e^	16.03 ± 0.11^f^

*Note:* Based on Tukey's test (*p* ≥ .05), same letters within the same columns means that values are not significantly different.

**TABLE 5:** Minimum inhibitory concentration (MIC) and minimum bactericidal concentration (MBC) of *Ziziphora tenuior* against foodborne pathogens evaluated by microdilution method (mean ± SD)

Bacteria	MIC (mg/ml)	MBC (mg/ml)
Gram‐positives		
*S. aureus*	1.25	1.25
*S. epidermis*	0.625	1.25
*L. monocytogenes*	0.625	0.625
*B. cereus*	1.25	2.5
*B. subtilis*	0.625	1.25
*E. faecalis*	1.25	2.5
Gram‐negatives		
*E. coli*	2.5	5
*S. typhimurium*	2.5	2.5
*K. pneumonia*	2.5	5
*S. dysenteriae*	1.25	2.5

Moreover, the results obtained in the current study were in conformity with the study of Shahbazi (2016), which reported that *S. epidermis, L. monocytogenes*, and *B. subtilis* were the most sensitive bacteria to *Z. clinopodioides* EO. The antimicrobial activities of *Z. clinopodioides* EO against some types of bacteria have also been reported in other studies (Behravan et al., 2007; Ozturk & Ercisli, 2007). Comparable results reported by these studies may be due to similar constituents of *Z. clinopodioides*.

The outcome obtained by MIC and MBC exposed that *B. subtilis*, *L. monocytogenes*, and *S. epidermis* were the most impressible bacteria to the ZEO, which had the lowest MIC. Conversely, *E. coli*, *S. typhimurium*, and *K. pneumonia* were the most resistant bacteria, and higher amounts of ZEO were needed to inhibit them. Among all Gram‐negative bacteria, *S. dysenteriae* was the most susceptible bacteria that has been inactivated by 1.25 mg/ml of ZEO. A number of studies reported the antimicrobial activity of ZEO (Dakah et al., 2019; Hazrati et al., 2020). The potent antibacterial properties of ZEO may be attributed to the high content of phenolic compound as well as monoterpenes such as pulegone, which was the main components of ZEO in this study. Although the action mechanisms of oxygenated monoterpenes have not been fully comprehended, there is evidence showing the interference with lipophilic compounds of the bacterial membrane (Behravan et al., 2007).

Overall, the data demonstrated that ZEO was effective against Gram‐positive bacteria more than Gram‐negative ones. This is in compliance with the study of Hamedi et al. (2017), which investigated the antibacterial activity of *Ziziphora persica* EO and reported lower MIC and MBC for Gram‐positive bacteria than Gram‐negatives. In addition, other studies also indicated reasonable antibacterial effect of ZEO (Hazrati et al., 2020; Nazemisalman et al., 2018). It has been suggested that the existence of a major layer of lipopolysaccharide and lipoprotein in the cell wall of these bacteria (Gram‐negative) makes them resistant to ZEO, while the thin layer of Gram‐positive bacteria is their weakness against EOs such as *Ziziphora* species EOs (Mahdavi et al., 2020).

### 3.5 Microbiological analysis of cooked sausage

**FIGURE 1:**
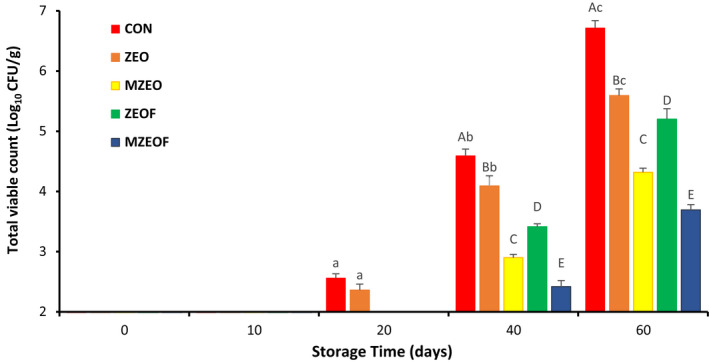
Effects of different treatments on total viable count (TVC) of cooked beef sausages during 60 days (mean ± SD). Based on Tukey's test (*p* ≥ .05), same capital letters within the same days and same small letters within the same samples means that values are not significantly different

**FIGURE 2:**
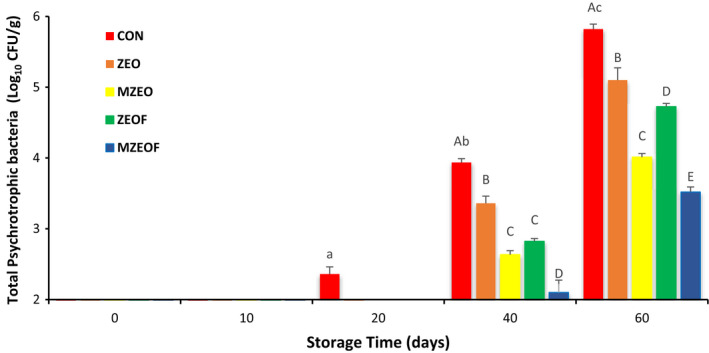
Effects of different treatments on total psychrophilic count (PSY) of cooked sausages during 60 days (mean ± SD). Based on Tukey's test (*p* ≥ .05), same capital letters within the same days and same small letters within the same samples means that values are not significantly different

**FIGURE 3:**
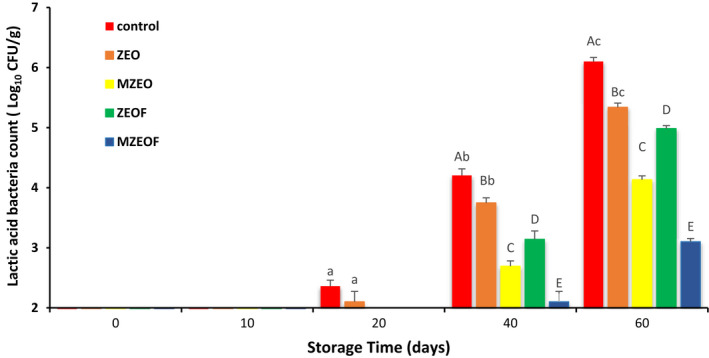
Effects of different treatments on lactic acid bacteria (LAB) of cooked sausages during 60 days (mean ± SD). Based on Tukey's test (*p* ≥ .05), same capital letters within the same days and same small letters within the same samples means that values are not significantly different

TVC, PSY, and LAB of cooked sausage for different treatments during 60 days of storage are outlined in Figures 1, 2, and 3, respectively. At the beginning of the storage period, no bacteria were detected in all groups, and similar results were observed on day 10 of storage. This phenomenon was in conformity with the study of Aminzare et al. (2018) that stated the same results. But on day 20, TVC and LAB values in the CON and ZEO groups, as well as PSY value in the CON group, were counted, and a gradual increase was observed in all studied groups until the last day of storage. At the final day, the highest bacterial count for all studied bacteria was observed in the control group, followed by ZEO, ZEOF, MZEO, and MZEOF (*p* ≤ .05). The highest value in TVC analysis was recorded for control group (6.71 log CFU/g), while the lowest amount was measured for MZEOF (3.69 log CFU/g). Similar results were measured on day 60 for PSY analysis (control: 5.81 and MZEOF: 3.51 log CFU/g). With regard to LAB, the highest bacterial count in day 60 was recorded for the control group (6.09 log CFU/g), and the lowest was for the MZEOF treatment (3.10 log CFU/g).

The results showed lower values for ZEOF in comparison with the ZEO group at day 60, indicating a significant effect of fiber addition on microbial counts of sausage (*p* ≤ .05). Moreover, according to ISIRI (standards institute of Iran), the maximum acceptable limit for TVC in cooked sausages is set at 5 log CFU/g (ISIRI, 2005). MZEO and MZEOF groups were the only samples that did not exceed this limit at day 60, demonstrating the favorable effect of ZEO microencapsulating on sausage microbial counts (*p* ≤ .05). The best results (lowest values) for microbiological analysis were observed in the MZEOF group, which contained both microencapsulated ZEO and orange fiber, simultaneously (*p* ≤ .05). The antimicrobial activity of encapsulated EOs and their impact on shelf life and microbial quality of meat products were investigated by researchers. Hadian et al. (2017) evaluated the application of encapsulated *Rosmarinus officinalis* EO in chitosan‐benzoic acid nanogel and stated that it significantly increased the shelf life of beef cutlet compared to free EO. Radünz et al. (2020) encapsulated thyme EO by using casein‐maltodextrin and investigated its effects on the shelf life of hamburger‐like meat products stored for 14 days. They reported that encapsulated thyme EO significantly improved the microbial quality and shelf life of meat products compared to other groups, including unencapsulated thyme EO. In another study, encapsulated clove EO was developed using sodium alginate and investigated for its antimicrobial and antioxidant effects as well as its impact on the shelf life of the meat‐like products (Radünz et al., 2019). The effect of microencapsulated *Z. clinopodioides* on the fish burgers was investigated by Shahinfar et al. (2017). They compared the antibacterial effects of free and microencapsulated *Z. clinopodioides* and reported that the microencapsulation process increased its antibacterial activity. It has been proven that microencapsulation of EOs can increase their desirable properties, such as antimicrobial activity (Bilenler et al., 2015; Piletti et al., 2019). This phenomenon (increased antibacterial activity after microencapsulation) may explain the better results of microencapsulated treatment (MZEO and MZEOF) in beef sausage samples compared to other groups.

Moreover, microencapsulation of EOs not only increases their antimicrobial activity, but also increases their duration of impact. Yang et al. (2021) showed that microencapsulation of cinnamon EO increased both antimicrobial properties and duration of effects. Also, based on the findings of our study, the addition of orange fiber in both samples containing free ZEO and microencapsulated ZEO caused a significant reduction in bacterial growth (*p* ≤ .05). This may be due to the indirect and mild effect of fiber addition on microbial suppression through reduction of water activity (Sayas‐Barberá et al., 2012). In addition, the presence of bioactive compounds in both ZEO and orange fiber, such as terpenes and polyphenols, can increase the antibacterial effects (Viuda‐Martos et al., 2010b). Overall, MZEOF was significantly lower than other treatments for TVC, PSY, and LAB tests (*p* ≤ .05), indicating the highest antimicrobial activity due to a combination of microencapsulation of ZEO and using orange fiber. The desirable antimicrobial effects of simultaneous addition of orange fiber to EOs in sausages were confirmed by other studies (Viuda‐Martos et al., 2010a, 2010b). Viuda‐Martos et al., 2010a added 1% orange fiber and different EOs (0.02% thyme or 0.02% rosemary) to *mortadella* for the purpose of investigating their impact on the shelf life of this sausage. They observed the best result for the combination of orange fiber and EOs, indicating the synergistic effect of this combination on bacterial growth. In another study by Viuda‐Martos et al. (2010b), oregano EO (0.02%) and 1% orange dietary fiber was added to bologna sausages, and the effect of this treatment was investigated. The results revealed oxidative stability and bacterial count reduction, as well as shelf life extension.

### 3.6 Evaluation of lipid oxidation in cooked sausage

In this study, PV test was done to evaluate the oxidation of lipids in sausage samples, and the constant increasing trend was observed for this analysis. Peroxide value is one of the most common analyses for the evaluation of primary oxidation in foods. The PV values at the beginning of the storage were between 1.38 meq/kg lipid and 1.49 meq/kg lipid (*p* ≥ .05), which were reached 10.41–27.30 meq/kg lipid (*p* ≤ .05) at day 60. The highest and lowest PV values were measured for control (27.30 meq/kg lipid) and MZEOF (10.41 meq/kg lipid) at day 60, respectively. The findings pointed out that microencapsulation of ZEO and the addition of orange fiber to sausage samples significantly reduced the amounts of PV values in comparison with the control group (*p* ≤ .05). Moreover, better outcomes were discovered in MZEOF treatment with the lowest amounts of PV values (*p* ≤ .05) in cooked beef sausages, regardless of the storage day. These data were compatible with the results of Hu et al. (2015), which reported lower PV values for pork samples treated with encapsulated cinnamon EO, compared to the control group. The encapsulation treatment may be responsible for better outcomes in the PV test. Other studies on the combination of EOs and fiber showed similar results to the current study. Viuda‐Martos et al. (2010b) evaluated the beneficial effects of the addition of orange fiber and/or oregano EO in different packaging environments, such as vacuum packaging, modified atmosphere, and air, on the quality and durability of bologna sausages. They stated that regardless of packaging conditions, the combination of oregano EO and orange fiber exhibited the best results in PV analysis. Similar results were observed for the combination of orange dietary fiber and thyme and rosemary EOs applied on *mortadella* to improve its shelf life and quality (Viuda‐Martos et al. (2010a)). The lower oxidation degree of lipids observed in our study for MZEOF treatment could be attributed to the protective effect of ZEO and orange fiber. Bioactive compounds possessing antioxidant properties in ZEO and orange fiber, such as polyphenols, may be responsible for the antioxidant effect.

The TBARS analysis was performed in this study to evaluate the secondary metabolites produced by oxidation of lipids in meat sausage samples during storage at 4°C, and the measured values are presented in Table 6. A constant increase in TBARS values was observed in sausage samples throughout the storage. The initial values of TBARS for all treatments were in the range of 1.18–1.25 mg MDA/kg sausage (*p* ≤ .05), which increased and reached a range of 3.10–5.11 mg MDA/kg (*p* ≤ .05) at day 60. Parallel to the results of the PV test, for TBARS analysis, the highest and lowest values at day 60 were measured for control (5.11 mg MDA/kg) and the MZEOF (3.10 mg MDA/kg), respectively. The results disclosed that the lowest values for TBARS analysis were observed in MZEOF treatment (*p* ≤ .05). However, microencapsulated ZEO and the addition of fiber were also able to significantly decrease TBARS values in MZEO and ZEOF groups (*p* ≤ .05). Hemmatkhah et al. (2020) encapsulated cumin seed EO to extend the shelf life of beef hamburgers. They reported that the amounts of TBARS in treatment hamburger samples were lower than the untreated group, which was compatible with the data obtained in the current study. Better results of TBARS analysis in MZEO and MZEOF may be ascribed to the gradual release of antioxidant agents from encapsulated ZEO—conducive to maintaining antioxidant activity for a longer period (Table 7).

**TABLE 6:** Effects of different treatments on peroxide value (meq/kg lipid) of cooked sausage during 60 days (mean ± SD)

Sample	Storage time (days)				
	0	10	20	40	60
CON	1.41 ± 0.12^Aa^	4.23 ± 0.12^Cb^	16.30 ± 0.14^Ac^	25.10 ± 0.14^Ad^	27.30 ± 0.13^Ae^
ZEO	1.38 ± 0.11^Aa^	2.73 ± 0.10^Bb^	11.74 ± 0.12^Bc^	20.08 ± 0.09^Bd^	22.12 ± 0.09^Be^
MZEO	1.49 ± 0.09^Aa^	2.15 ± 0.09^Ab^	6.33 ± 0.09^Cc^	11.49 ± 0.12^Cd^	12.54 ± 0.10^Ce^
ZEOF	1.45 ± 0.13^Aa^	2.62 ± 0.11^Bb^	9.85 ± 0.13^Dc^	16.43 ± 0.13^Dd^	17.59 ± 0.11^De^
MZEOF	1.42 ± 0.10^Aa^	2.05 ± 0.12^Ab^	5.09 ± 0.12^Ec^	9.63 ± 0.12^Ed^	10.41 ± 0.10^Ee^

*Note:* Based on Tukey's test (*p* ≥ .05), same capital letters within the same columns and same small letters within the same rows means that values are not significantly different.

**TABLE 7:** Impact of different treatments on TBARS values (mg MDA/kg) of cooked sausage during 60 days (mean ± SD)

Sample	Storage time (days)				
	0	10	20	40	60
CON	1.22 ± 0.070^Aa^	1.92 ± 0.085^Db^	2.45 ± 0.075^Cc^	3.64 ± 0.07^Dd^	5.11 ± 0.08^Ae^
ZEO	1.18 ± 0.075^Aa^	1.70 ± 0.075^Cb^	2.18 ± 0.065^Bc^	3.22 ± 0.065^Cd^	4.4 ± 0.075^Be^
MZEO	1.20 ± 0.065^Aa^	1.48 ± 0.065^ABb^	1.80 ± 0.07^Ac^	2.54 ± 0.08^Ad^	3.39 ± 0.075^Ce^
ZEOF	1.25 ± 0.07^Aa^	1.66 ± 0.05^BCb^	2.03 ± 0.09^Bc^	3.12 ± 0.08^Cd^	4.11 ± 0.08^De^
MZEOF	1.18 ± 0.08^Aa^	1.42 ± 0.05^Ab^	1.67 ± 0.08^Ac^	2.20 ± 0.07^Bd^	3.10 ± 0.07^Ee^

*Note:* Based on Tukey's test (*p* ≥ .05), same capital letters within the same columns and same small letters within the same rows means that values are not significantly different.

The reduction of TBARS values may be due to multiple factors. First, various fibers, including orange fiber, containing various bioactive compounds such as polyphenols can reveal antioxidant effects. These bioactive compounds act as a potent antioxidant and scavenge the hydroxyls and free radicals. Moreover, polyphenols can play a role as metal chelators and inhibitors of enzymatic reactions initiating the oxidation processes (Fernández‐López et al., 2007; Viuda‐Martos et al., 2010b). Second, the presence of essential oils could significantly reduce the TBARS values. Third, the encapsulation of essential oil could extend the duration of the antioxidant effect of essential oils. Fourth, the excellent effect of combination of EOs and fibers on TBARS analysis should be taken into consideration which has been reported by Viuda‐Martos et al. (2010b) and Viuda‐Martos et al. (2010a) in bologna sausages and *mortadella*, respectively.

## 4 CONCLUSION

In this study, 0.5% free or microencapsulated ZEO, alone and in combination with 1% orange fiber, was added to the formulation of cooked beef sausage, and their impacts on microbial (TVC, PSY, and LAB) and chemical (PV and TBARS) qualities of the product were assessed. The initial evaluation of ZEO composition and properties showed acceptable potential for application in the sausage. The major constituent of ZEO was pulegone (47.12%), followed by isomenthone (14.57%). The data obtained by microbiological and lipid oxidation corroborated that all treatment groups were able to maintain the properties of sausage. Considering this, the best results were observed in the sausage samples treated with 0.5% microencapsulated ZEO and 1% orange fiber (MZEOF) at the end of the storage period [TVC (3.69 log CFU/g), PSY (3.51 log CFU/g), LAB (3.1 log CFU/g), PV (10.41 meq/kg lipid), and TBARS (3.1 mg MDA/kg)]. Overall, our study suggests that the combination of microencapsulated ZEO and orange fiber can be used in processed meat product to extend the shelf life.

## CONFLICT OF INTEREST

None.
